# Baicalin Prevents Chronic β‐AR Agonist‐Induced Heart Failure via Preventing Oxidative Stress and Overactivation of the NADPH Oxidase NOX2

**DOI:** 10.1111/jcmm.70388

**Published:** 2025-02-23

**Authors:** Yixuan Ge, En Ma, Xiaowei Guo, Qing Wang, Weidong Zhu, Dan‐ni Ren, Da Wo

**Affiliations:** ^1^ Academy of Integrative Medicine, College of Integrative Medicine, Fujian Key Laboratory of Integrative Medicine on Geriatric Fujian University of Traditional Chinese Medicine, Fujian Key Laboratory of Integrative Medicine on Geriatric Fuzhou Fujian China

**Keywords:** baicalin, heart failure, isoproterenol, NADPH oxidase, β‐ARs

## Abstract

Heart failure (HF) remains the leading cause of mortality worldwide. Although various drugs are currently used in the treatment of HF, including angiotensin receptor blockers, angiotensin‐converting enzyme inhibitors and beta blockers, none of these drugs can reverse the physiological remodelling of the heart associated with HF. Therefore, discovering novel drugs that can limit the extent of HF or prevent the structural dysfunction of the heart during HF progression is urgently needed. Baicalin is a natural flavonoid widely used in Traditional Chinese Medicine for its anti‐inflammatory and anti‐oxidative effects; however, the role of baicalin in chronic HF, in particular its underlying mechanisms of action, remains largely unelucidated. Murine models of beta‐adrenergic receptor agonist (β‐AR)‐induced HF were induced via chronic induction with isoproterenol (ISO) for 4 weeks. Furthermore, we examined the effects and mechanisms of baicalin in protecting against ISO‐induced cardiac impairment and HF. Daily administrations of baicalin robustly protected against chronic ISO‐induced pathophysiological changes of the heart, including cardiac hypertrophy, reduced ejection fraction, fibrosis and remodelling. Baicalin also strongly inhibited the production of reactive oxygen and nitrogen species in the heart by preventing overactivation of the NADPH oxidase NOX2. Hence, the cardioprotective effects of baicalin in preventing chronic β‐AR‐induced HF were due to preventing the overactivation of NOX2 and generation of excessive oxidative stress. Our findings provide new mechanistic insight and suggest the therapeutic potential of baicalin as a novel drug in the treatment of chronic HF.

## 1 Introduction

Heart failure (HF) remains the leading cause of mortality and morbidity worldwide, with a 5‐year mortality rate of 50%–70%, and despite recent advancements in clinical treatment options, the number of people with HF has continued to increase [1, 2]. HF is characterised by a structural or functional abnormality of the heart that results in reduced cardiac output and/or elevated intracardiac pressure, ultimately leading to death [3]. Patients with HF are often comorbid with disorders such as ischaemic heart disease including myocardial infarction, hypertension or other cardiovascular diseases. Although there are a wide variety of drugs that are currently used in the treatment of HF, most notably angiotensin receptor blockers, angiotensin‐converting enzyme inhibitors, beta blockers and mineralocorticoid receptor antagonists [1], none of these drugs can actually reverse HF or the physiological remodelling of the heart associated with HF. Therefore, the discovery of novel drugs that can not only prevent the worsening of HF but also provide new mechanisms in treating the structural or functional dysfunction of the heart may be urgently needed.

One of the main causes of HF is the chronic hyperactivation of cardiac beta‐adrenergic receptors (β‐ARs), which are G‐protein‐coupled receptors (GPCRs) that mediate the sympathetic adrenergic effect on the heart. There are three β‐AR subtypes that are expressed in the heart: β_1_‐AR, β_2_‐AR and β_3_‐AR, with β1‐ARs being the predominant subtype, accounting for approximately 80% [4]; β1‐ and β2‐ARs are the main stimulatory receptors of cardiac function [5] and overactivation of these β‐ARs results in significantly worsened pathological remodelling and function of the heart.

Isoproterenol (ISO) is a synthetic sympathomimetic amine with structural similarities to epinephrine and binds almost exclusively to β‐ARs [6], and hence the overactivation of β‐ARs using ISO is used extensively in animal models for modelling HF. When ISO binds to β‐ARs, there is a rapid increase in cardiac contractility and cardiac output, which is beneficial in the short term. However, sustained long‐term sympathetic stimulation of ISO results in the constant activation of β‐ARs that leads to numerous pathophysiological changes in the heart such as cardiac remodelling, hypertrophy, fibrosis, reduced ejection fraction (EF) and ultimately HF. The body compensates for the chronic stimulation of β‐ARs via the process of receptor internalisation, which results in a decrease in β‐AR receptors in the plasma membrane [4, 7].

Baicalin is a natural flavonoid isolated from the roots of the perennial herb *Scutellaria baicalensis* Georgi [8] that has been widely used in Traditional Chinese Medicine due to its beneficial effects in various diseases, including cardiovascular diseases [9, 10, 11, 12]. Baicalin exhibits numerous pharmacological effects, including anti‐inflammatory, anti‐oxidative and immune‐boosting properties. However, the role of baicalin in chronic β‐AR‐induced HF, in particular its underlying mechanisms of action, remains largely unelucidated. In this study, we aimed to investigate the potential cardioprotective effects of baicalin in protecting against chronic β‐AR‐induced HF.

## 2 Materials and Methods

### 2.1 Drugs and Reagents

Reagent grade baicalin (CAS number: 21967‐41‐9; purity: 99.17%) was purchased from Shanghai Haoyuan Biotechnology (China). Baicalin was dissolved in dimethyl sulfoxide (DMSO) to a stock concentration of 100 mg/mL and stored at −80°C until further use. Reagent grade ISO was purchased from Sigma Aldrich (USA). For all experiments, baicalin was administered by intraperitoneal (i.p.) injection (100 mg/kg/day), while a chronic model of HF was induced by daily subcutaneous (s.c.) injections of ISO (15 mg/kg/day).

### 2.2 Animal Studies

All procedures were performed in accordance with the Guide for the Care and Use of Laboratory Animals published by the National Institutes of Health, complied with the ARRIVE guidelines and were approved by the Experimental Animal Care and Use Committee of Fujian University of Traditional Chinese Medicine (No. FJTCM IACUC 2023025). Male C57BL/6 mice (8–12 weeks of age) weighing 25–27 g were purchased from Shanghai SLAC Laboratory Animal Co. Ltd. (Shanghai, China). Subsequently, mice were divided into three groups: (1) Control group (PBS administered as vehicle); (2) ISO model group (15 mg/kg/day, s.c.); (3) ISO + Baicalin treatment group (100 mg/kg/day, i.p.). For both model and treatment groups, baicalin was administered at least 2 h prior to ISO administration, and equal volumes of either ISO or PBS as vehicle were administered to mice in each group. Mice were killed at 4 weeks by euthanasia via an overdose of sodium pentobarbital (200 mg/kg) injected intraperitoneally.

### 2.3 Echocardiographic Assessment

Cardiac function was assessed using the Vevo 2100 echocardiography imaging system (Visual Sonics, Canada). Mice were anaesthetised using 2% isoflurane supplemented with oxygen, and heart rate was strictly controlled between 400 and 450 bpm. M‐mode measurements were used to determine parameters of left ventricular (LV) systolic function, including left ventricular internal diameter at systole (LVID;s), left ventricular internal diameter at diastole (LVID;d), as well as left ventricular ejection fraction (LVEF%) and fractional shortening (LVFS%).

### 2.4 Western Blot Analysis

Western blot analysis was performed according to a standard protocol. Briefly, following killing, mice hearts were carefully harvested and snap‐frozen in liquid nitrogen. Heart lysate total proteins were extracted using RIPA lysis buffer (Beyotime Biotech, China). Equal concentrations of proteins were loaded onto SDS‐PAGE gel for electrophoresis, transferred onto a 0.22‐μm polyvinylidene difluoride (PVDF, Merck Millipore, MA, USA) membrane, blocked with 5% nonfat dried milk and then incubated with the indicated primary antibodies overnight at 4°C. The following antibodies were used: ANP (Abcam, ab225844), BNP (Abcam, ab236101), SOD (PTM Bio, PTM‐5681), Catalase (PTM Bio, PTM‐5630), PRDX1 (PTM Bio, PTM‐5367), α‐actin (Proteintech, 66125‐1‐Ig). Subsequently, membranes were incubated with the respective secondary antibodies, and the resulting protein bands were detected via chemiluminescence. Quantification of blots was performed using ImageJ software.

### 2.5 Histological Staining

Haematoxylin and eosin (H&E) and Masson's trichrome staining were performed according to a standard protocol. Briefly, mouse hearts were fixed in 4% paraformaldehyde for 24 h and washed using tap water overnight before dehydration using an ethanol gradient. Subsequently, tissues were cleared in xylene, paraffin‐embedded, then prepared into 6‐μm‐thick sections and stained with H&E to measure the myocyte cross‐sectional area, or Masson's trichrome to determine the extent of myocardial fibrosis. Finally, stained sections were visualised via a light microscope (Leica, Germany).

### 2.6 Quantitative Real‐Time PCR

Quantitative real‐time PCR was performed according to a standard protocol. Briefly, the total RNA was extracted from frozen heart tissues using TRIzol reagent and reverse transcribed to cDNA using a PrimeScript RT reagent Kit according to the manufacturer's instructions (Takara, Japan). qPCR was performed with SYBR Green master mix in 384‐well optical plates using a QuantStudio 7 Flex RealTime PCR System (Applied Biosystems, USA). The primer sequences used for amplification were as follows: Collagen I (Forward: 5′‐ATGGATTCCCGTTCGAGTAC‐3′, Reverse: 5′‐TCAGCTGGATAGCGACATCG‐3′), Collagen III (Forward: 5′‐CGTAGATGAATTGGGATGCA‐3′, Reverse: 5′‐ACATGGTTCTGGCTTCCAG‐3′).

### 2.7 Immunofluorescence Staining

For immunofluorescence staining, freshly extracted hearts were embedded in Tissue‐Tek OCT compound and frozen prior to sectioning. Slides were rinsed with PBS and then fixed with 4% paraformaldehyde for 30 min, permeabilised with 0.25% Triton X‐100, blocked with 5% bovine serum albumin (BSA), and incubated with the respective primary antibodies at 4°C overnight. Subsequently, slides were incubated with the respective Alexa Fluor‐conjugated secondary antibodies (Abcam, USA) and mounted in antifade slide mounting media. Images were acquired using laser confocal scanning microscopy (Zeiss LSM 710, Germany).

### 2.8 Molecular Docking

Molecular docking was performed by AutoDock Vina version 1.1.2 and AutoDockTools (ADT). The molecular structure of baicalin was retrieved from PubChem Compound. The predicted structures of NOX2 (Mouse: AF‐Q3U6G0‐F1, Human: AF‐P04839‐F1) were downloaded from AlphaFold (https://alphafold.ebi.ac.uk/). The X‐ray crystal structure of the baicalin –NOX2 complex was downloaded from PDB (http://www.rcsb.org/). For docking analysis, all protein and molecular files were converted into PDBQT format with all water molecules excluded and polar hydrogen atoms added. Molecular docking and determination of binding affinities were performed by AutoDock Vina 1.1.2 and visualised using the PyMOL (version 2.5.0) molecular graphics system.

### 2.9 Statistical Analysis

All data are presented as mean ± SEM. Statistical analysis was performed using SPSS 28.0 software (Chicago, IL, USA). Student's *t*‐test or one‐way ANOVA with Fisher's LSD multiple comparison test was performed to compare differences between two or greater than two groups, respectively. *p* < 0.05 was considered statistically significant.

## 3 Results

### 3.1 Baicalin Improves Cardiac Function and Prevents Chronic β‐AR Agonist‐Induced Heart Failure

**FIGURE 1:**
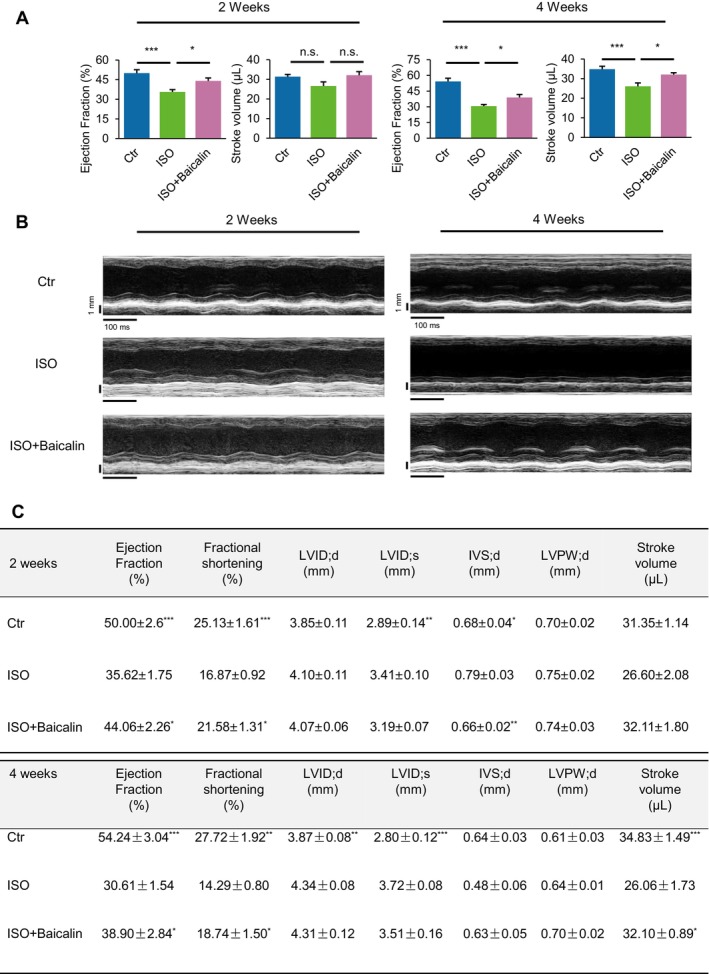
Baicalin protects against chronic beta‐adrenergic receptor (β‐AR) agonist‐induced heart failure. (A–C) Cardiac function parameters including left ventricular (LV) ejection fraction (EF %) and stroke volume (A), representative M‐mode echocardiography showing LV end‐systolic and end‐diastolic dimensions and LV wall motion (B), and table summarising echocardiography parameters of mice (C) following chronic induction of β‐AR agonist (isoproterenol [ISO]) for 2 and 4 weeks. Data are presented as mean ± SEM. n.s., not significant; *p < 0.05, **p < 0.01, ***p < 0.001, *n* = 5 or more for each group. LVID;d/LVID;s, left ventricular internal dimension at diastole/systole; IVS;d, interventricular septum thickness at diastole; LVPW;d, LV posterior wall thickness at diastole.

We first examined the potential effects of baicalin in chronic HF by daily administrations of a general βAR agonist, ISO. Cardiac function was assessed via echocardiography, which showed significant reductions in LVEF and LVFS parameters from 2 weeks following ISO administration, which was further worsened at 4 weeks (Figure 1A). It is interesting to note that at 2 weeks post‐ISO induction, the LV wall thickness was actually slightly increased, indicating an early compensatory response of cardiac hypertrophy, but which was significantly decreased at 4 weeks, indicative of a decompensatory response and occurrence of HF with overt systolic dysfunction (Figure 1A,B). Notably, mice administered baicalin exhibited significant improvements in cardiac function parameters as well as LV myocardial wall movement and thickness compared to PBS‐administered mice following ISO induction (Figure 1A,B). The summary of these cardiac function parameters is outlined in Figure 1C.

### 3.2 Baicalin Decreases the Extent of Cardiac Hypertrophy and Fibrosis in HF

**FIGURE 2:**
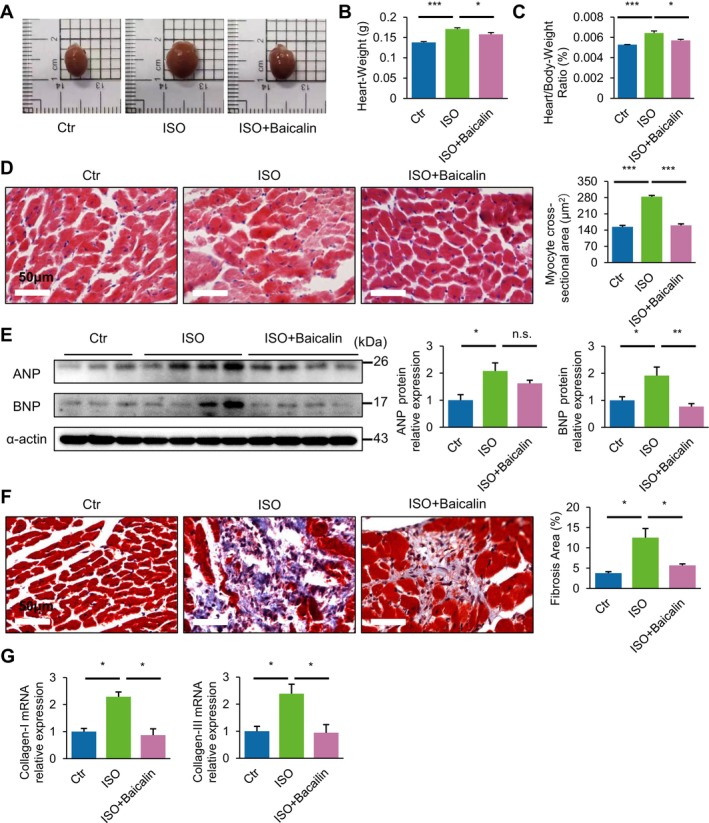
Baicalin prevents cardiac hypertrophy and fibrosis in heart failure. (A–G) Representative images of excised heart (A), heart weight (B), heart weight/body weight ratio (C), representative H&E‐stained sections and quantification of estimated myocyte cross‐sectional area (D), representative immunoblots and densitometry analysis of heart failure markers atrial natriuretic peptide (ANP) and brain natriuretic peptide (BNP) (E), representative Masson's trichrome‐stained sections (red, muscle fibres; blue, collagen‐rich fibrotic regions) and quantification of estimated intraventricular fibrosis (F), mRNA expression levels of myocardial fibrosis markers of Col‐I and Col‐III (G) following chronic induction of beta‐adrenergic receptor agonist (isoproterenol) for 4 weeks. Data are presented as mean ± SEM. n.s., not significant; *p < 0.05, **p < 0.01, ***p < 0.001; *n* = 3 or more in each group; scale bar, 50 μm.

One of the key characteristics of HF is cardiac hypertrophy, which consists of concentric in HF with preserved EF, or eccentric in HF with reduced EF. In the current model of chronic ISO‐induced HF, there were significant increases in heart size as observed at 4 weeks post‐model, and heart weight to body weight ratio (Figure 2A–C). In addition, the cross‐sectional area of cardiomyocytes was markedly increased following ISO induction, as visualised via H&E staining (Figure 2D), indicative of a significant degree of cardiac hypertrophy occurrence. Notably, mice that were administered baicalin significantly attenuated the ISO‐induced increases in heart weight and cardiomyocyte size (Figure 2A–D). Furthermore, key markers of HF including atrial natriuretic peptide (ANP) and brain natriuretic peptide (BNP), which were also increased in ISO‐induced HF, were significantly rescued by treatment with baicalin (Figure 2E).

Another key feature in the pathogenesis of failure hearts is the occurrence of cardiac fibrosis, whereby healthy myocardium is replaced by fibrotic scar tissue. Masson's trichrome staining of the LV myocardium exhibited significant areas of cardiac fibrosis in chronic ISO‐induced failure hearts surrounding the endocardial wall, compared to baicalin‐treated mice hearts that had markedly lower fibrosis, and negligible levels of control hearts (Figure 2F). Moreover, real‐time PCR analysis of key fibrosis markers collagen‐I and collagen‐III was significantly increased more than twofold in the LV myocardium of ISO‐treated mice at 4 weeks, which was markedly attenuated in mice treated with baicalin (Figure 2G). Taken together, these results demonstrate that baicalin significantly alleviates chronic ISO‐induced cardiac hypertrophy and fibrosis in HF.

### 3.3 Baicalin Prevents Excessive Production of Reactive Oxygen and Nitrogen Species

**FIGURE 3:**
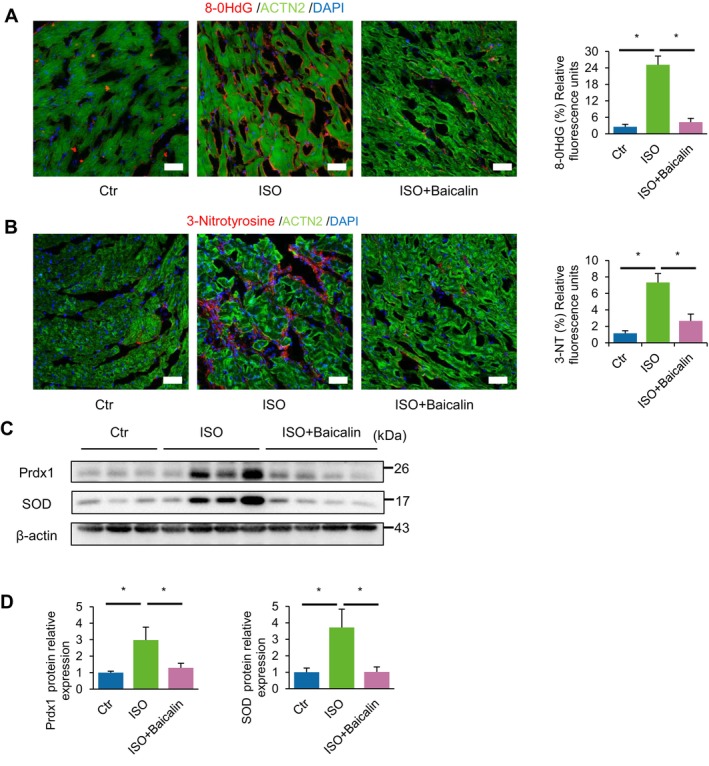
Baicalin decreases oxidative stress‐induced myocardial damage. (A, B) Representative immunofluorescence staining of oxidative damage marker 8‐OHdG positive foci (A, red) and reactive nitrogen species marker 3‐nitrotyrosine (3‐NT) positive foci (B, red), as well as quantification of relative fluorescence (right panels), cardiac muscle marker ACTN2 (green), and DAPI (blue). (C, D) Representative immunoblots (C) and densitometry analysis (D) of antioxidant enzymes peroxiredoxin‐1 (Prdx1) and superoxide dismutase (SOD) expression following chronic induction of beta‐adrenergic receptor agonist (isoproterenol [ISO]) for 4 weeks. Data are presented as mean ± SEM. n.s., not significant; *p < 0.05; *n* = 3 or more in each group; scale bar, 50 μm.

Oxidative stress is a key phenomenon that occurs during various stages of cardiac dysfunction, including HF, via excessive production of reactive oxygen species (ROS) or reactive nitrogen species (RNS) that can cause and exacerbate cardiomyocyte damage [13]. We performed immunofluorescence staining in the myocardium of mice subjected to chronic injections of ISO for 2 and 4 weeks, and observed significantly more positively stained sections of both 8‐OHdG (a marker of ROS and DNA damage occurrence) and 3‐nitrotyrosine (3‐NT; a marker of RNS occurrence), demonstrating that chronic activation of β‐ARs by ISO causes significant oxidative stress to the heart (Figure 3A,B). Notably, mice administered baicalin significantly reduced myocardial levels of both 8‐OHdG and 3‐NT following ISO induction, demonstrating the robust anti‐oxidative ability of baicalin in preventing chronic ISO‐induced oxidative stress in HF (Figure 3A,B).

Excessive oxidative stress further overloads the body's innate ability to effectively neutralise the ROS/RNS via activation of antioxidant enzymes, which results in an imbalance in the endogenous antioxidant defence system [13]. We further performed Western blot analyses, which showed that ISO‐administered mice indeed showed significant elevation in the expression of key antioxidant enzymes catalase, peroxiredoxin‐1 (PRDX1) and superoxide dismutase (SOD) in the myocardium following 4 weeks (Figure 3C,D), further confirming that chronic ISO induction results in excessive production of ROS. Of note, baicalin‐administered mice markedly attenuated these ISO‐induced elevations of antioxidant enzymes, which further supported the fact that baicalin treatment prevented excessive production of ROS/RNS, thereby decreasing the extent of myocardial oxidative stress in chronic HF.

### 3.4 Baicalin Inhibits ROS/RNS by Preventing Overactivation of the NADPH Oxidase NOX2

**FIGURE 4:**
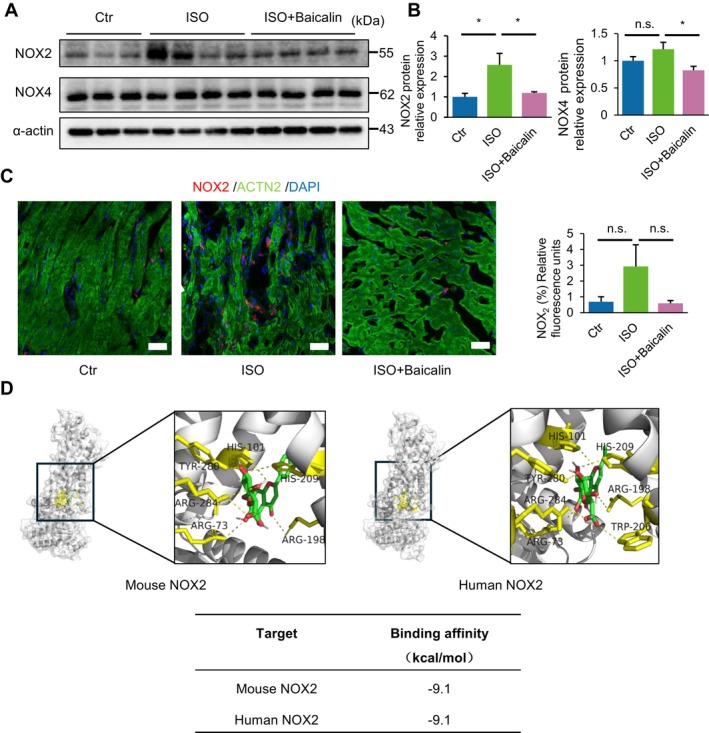
Baicalin inhibits reactive oxygen and nitrogen species via preventing overactivation of NOX2. (A–C) Representative immunoblot (A) and densitometry analysis (B) of NADPH oxidases NOX2 and NOX4 expression. Immunofluorescence staining showing NOX2‐positive foci (C, red), cardiac muscle marker ACTN2 (green) and DAPI (blue), and quantification of relative fluorescence (right panel) following chronic induction of beta‐adrenergic receptor agonist (isoproterenol [ISO]) for 4 weeks. (D) Molecular docking study on the interaction and predicted binding affinity between baicalin with NOX2 in both human and mouse. The 3D model structures were predicted using AlphaFold3 software. Data are presented as mean ± SEM. n.s., not significant; *p < 0.05; *n* = 3 or more in each group; scale bar, 50 μm.

We further examined the underlying mechanism of baicalin in preventing chronic ISO‐induced HF. Of note, the NADPH oxidase (NOX) family of membrane proteins are well known to be critically involved in the generation of superoxides, and hence is the major source of ROS and oxidative stress during numerous disease processes. In our current model of chronic ISO‐induced HF, we examined the protein expressions of NOX2 and NOX4, the two major isoforms expressed in the heart [14]. Western blot analysis showed that there was a significant increase in the level of NOX2 but not NOX4 expression in the myocardium of mice following chronic injection of ISO for 4 weeks (Figure 4A,B). Of note, mice administered baicalin significantly attenuated the increase in NOX2 levels following ISO induction, suggesting a decrease in the levels of endogenous superoxides generated (Figure 4A,B). Further, immunofluorescence staining showed markedly greater numbers of NOX2 foci in the myocardium of ISO‐induced mice, which was also significantly decreased in baicalin‐administered mice (Figure 4C).

Next, we examined the potential mechanism of baicalin in preventing excessive production of ROS/RNS by assessing the strength and nature of interactions between baicalin and NOX2 via molecular docking. Interestingly, baicalin exhibited a very high binding affinity of  −9.1 kcal/mol with the extracellular domain of both human and mouse NOX2 at the indicated sites (Figure 4D), suggesting that baicalin can strongly bind to and inhibit the function of NOX2 in the production of superoxides and ROS during chronic induction of ISO. Taken together, these findings demonstrate that baicalin inhibits oxidative stress in chronic ISO‐induced HF by binding to and preventing overactivation of the NADPH oxidase NOX2.

## 4 Discussion

One of the major functional abnormalities associated with HF is the occurrence of pathological ventricular remodelling, which mainly consists of LV hypertrophy, myocardial fibrosis and adverse changes in cardiac function [15, 16]. Thus, ventricular remodelling is often viewed as a compensatory mechanism, whereby the remaining cardiomyocytes become enlarged or exert improved functional capabilities in order to compensate for the decreased cardiac capacity [17]. Therefore, cardiac remodelling may be a double‐edged sword, whereby in the early stages of cardiac dysfunction, it is beneficial in order to maintain heart function; however, over time, there is often a progressive decline in heart function, eventually resulting in HF [18]. These phenotypes were also observed in our current study, where we utilised constant and chronic inductions of ISO as a β‐AR agonist similar to epinephrine, in order to model HF in vivo. Furthermore, because current β‐AR blocker type drugs cannot reverse HF or the physiological remodelling of the heart associated with HF, we aimed to determine the protective effects of baicalin as a potential novel drug that can assist in the treatment of HF.

Numerous studies have shown that the excessive generation of ROS that occurs during cardiac dysfunction is also a key factor that can lead to HF [19]. In addition, chronic activation of β‐ARs by ISO also causes extensive oxidative stress and leads to irreversible damage to the heart [20]. Hence, the inhibition of oxidative stress may be a key mechanism in treating or preventing the pathophysiological remodelling of the heart associated with HF. In this regard, baicalin has long been used in Traditional Chinese Medicine as a natural bioactive compound that has been shown to exhibit strong anti‐oxidative and anti‐inflammatory effects in various cardiovascular diseases [21, 22, 23], and hence baicalin may be a promising therapeutic agent for the treatment of HF. Our current study demonstrating that baicalin prevents excessive production of ROS and decreases the extent of myocardial oxidative stress further suggests its potential effects in the treatment of chronic HF.

Additionally, one of the major factors involved in the generation of ROS is via the NOX family of proteins. Although the NOX family members exhibit different modes of activity during various disease processes, they have shared roles in the production of hydrogen peroxide from superoxide anions, with the end result of causing oxidative stress. In our current model of chronic ISO‐induced HF, we found that NOX2 and NOX4, the two major isoforms expressed in the heart, had different trends of expression, whereby only NOX2 protein levels were significantly elevated following chronic ISO induction. We postulate that this may be because NOX2 is located on the plasma membrane, whereas NOX4 is located intracellularly, and hence NOX2 can bind various agonists for increasing or decreasing its expression, as well as the acute activation of superoxide anions [24]. Hence, this is also the reason why administration with baicalin can bind to and significantly decrease the level of NOX2 expression in the plasma membrane, thereby inhibiting the overactivation and function of NOX2 in generating excessive ROS during ISO‐induced HF.

These findings elucidate the protective effects of baicalin in chronic HF by inhibiting myocardial oxidative stress and ROS production by preventing overactivation of the NADPH oxidase NOX2. These findings provide important insights into the role and mechanism of baicalin and suggest the potential use of baicalin as a novel drug in the treatment of HF.

## Author Contributions

**Yixuan Ge:** formal analysis (equal), investigation (lead), visualization (lead), writing – original draft (equal). **En Ma:** validation (equal), visualization (equal), writing – original draft (equal). **Xiaowei Guo:** resources (equal), visualization (equal). **Qing Wang:** resources (equal), visualization (equal). **Weidong Zhu:** resources (equal), supervision (equal). **Dan‐ni Ren:** funding acquisition (equal), project administration (equal), supervision (equal). **Da Wo:** conceptualization (lead), funding acquisition (equal), project administration (lead), supervision (lead), writing – review and editing (lead).

## Conflicts of Interest

The authors declare no conflicts of interest.

## Data Availability

The data that support the findings of this study are available from the corresponding author upon reasonable request.
